# Test–Retest Reliability and Convergent Validity of Two Scoring Versions of the Spinal Appearance Questionnaire Against Radiographic Measurements and Established Quality of Life Questionnaires in Adolescents with Idiopathic Scoliosis

**DOI:** 10.3390/children13010087

**Published:** 2026-01-06

**Authors:** Malik Alanazi, Eric C. Parent, Douglas P. Gross, Josette Bettany-Saltikov, Aislinn Ganci, Sarah Southon Hryniuk, Andrea Lin

**Affiliations:** 1 Department of Physical Therapy, Faculty of Rehabilitation Medicine, University of Alberta, 2-50 Corbett Hall, Edmonton, AB T6G 2G4, Canada; 2 Department of Physical Therapy and Health Rehabilitation, Prince Sattam Bin Abdulaziz University, Al-Kharj 11942, Saudi Arabia; 3 Department of Surgery, Faculty of Medicine and Dentistry, University of Alberta, 2D, 8440-112 Street Walter Mackenzie Health Sciences Centre, Edmonton, AB T6G 2B7, Canada; 4 Independent Consultant in Research Methods and Spinal Deformities, Stockton-on-Tees, Durham TS1 3BX, UK

**Keywords:** scoliosis, reproducibility of results, validation study, patient reported outcome measures, adolescent, quality of life, perceived appearance

## Abstract

**Highlights:**

**What are the main findings?**

- The SAQv1.1 test–retest reliability meets standards for research and individual use.
- All SAQv1.1 domains showed convergent validity with the SRS-22r and the Cobb angle and fewer ceiling effects compared to the original SAQ scores.

**What are the implications of the main findings?**

- The SAQv1.1 is recommended over the original SAQ in clinical and research settings.
- With smaller measurement error and fewer ceiling effects, the SAQv1.1 may help detect the effects of treatments for patients with small and moderate scoliosis curves.

**Abstract:**

**Background/Objectives**: The Spinal Appearance Questionnaire (SAQ) assesses the self-perception of appearance of adolescents with idiopathic scoliosis (AIS). Due to originally scoring too many domains and producing prevalent ceiling effects when treated conservatively, the SAQv1.1, has been introduced. This study aimed to compare the test–retest reliability, convergent validity, and ceiling and floor effects of the two versions of the SAQ in patients with AIS. **Methods**: Conservatively treated females with AIS, aged 10–18 years old, were consecutively recruited from a scoliosis clinic. The Scoliosis Research Society-22 refined (SRS-22r), SAQ, and SAQv1.1 were collected, in English, with radiographic measurements (Cobb angle, Coronal Balance, and Vertebral Rotations). Nine domain scores were obtained from the original SAQ. Appearance, Expectations, and Total scores were calculated for SAQ v.1.1. Questionnaires were re-administered electronically after one week. **Results**: One hundred females, aged 13.9 ± 1.8 years with curve angles of 28.8° ± 13.9°, were included. The test–retest reliability for SAQ varied between domains (ICC_3,1_ = 0.72 to 0.94). The Total, Appearance, and Expectation ICCs_3,1_ for the SAQv1.1 were 0.92, 0.94, and 0.86, respectively. Convergent validity was demonstrated between seven SAQ domains and the SRS-22r Total and Cobb angle (|r| = 0.32 to 0.59). The SAQv1.1 Total correlated with the SRS-22r Total (r = −0.50) and with the Cobb angle (r = 0.56). All SAQ domains presented ceiling (Curve = 11% to Kyphosis = 68%) and floor effects (Chest = 8% and Waist = 4%). The SAQv1.1 Total and Appearance had low ceiling effects (≤5%), while Expectations presented both ceiling (14%) and floor effects (10%). **Conclusions**: The SAQv1.1 is recommended because of its stronger reliability, superior convergent validity, and fewer ceiling and floor effects in AIS.

## 1. Introduction

Health-related quality of life (HRQOL) is the effect of a disease or a treatment on physical, psychological, and social domains of functioning and well-being [1]. HRQOL is influenced by scoliosis-related changes to appearance [1]. Appearance is among the reasons why patients with AIS undergo surgery [2]. Scoliosis-related changes in appearance may also lead to depression and low self-esteem [2].

The SRS-22 HRQOL questionnaire was developed for severe cases undergoing surgery [3]. However, its ability to capture the impact of smaller curves treated conservatively is severely limited [4,5], with nine items presenting major and another eleven having moderate ceiling effects [4]. Ceiling effects hinder the SRS-22’s ability to detect improvements and deteriorations in milder curves [4,5,6]. The Scoliosis Research Society has identified finding improved HRQOL assessments as a research priority because the SRS-22 is inadequate for monitoring the full continuum of care and severity in AIS.

The SAQ assesses perception of appearance as viewed from the back [7]. Sanders et al. modified the Walter Reed Visual Assessment Scale (WRVAS) [8] to develop the SAQ by adding four items and questions regarding self-image expectations [7]. The SAQ produces nine domain scores: General, Curve, Prominence, Trunk Shift, Waist, Shoulders, Kyphosis, Chest, and Surgical Scar. However, Mulcahey et al. interviewed adolescents and noted concerns with the SAQ’s complex terminology, vague questions, and illustrations that were challenging to interpret [9]. Moreover, Schreiber et al. reported ceiling effects over 27% for the SAQ, limiting the detection of changes in smaller curves treated with exercises [10].

Carreon et al. later created the Spinal Appearance Questionnaire version 1.1 (SAQv1.1) by modifying the SAQ, informed by factor analysis. The SAQv1.1 includes 14 items [11]. The first 10 items produce the Appearance score while the last 4 items produce an Expectations score. The SAQv1.1 also allows computing a Total score by combining both domains.

The sample studied by Carreon et al. while developing the SAQv1.1 presented an average curve severity deemed severe (55.8° ± 13.7°) [11]. Therefore, there is still a need to compare the measurement properties of the SAQ and the SAQv1.1 in patients with smaller curves treated non-operatively. This is necessary to show whether they work to monitor the full spectrum of scoliosis. This study aims to compare the test–retest reliability and convergent validity of these two SAQ versions in AIS. The secondary objective is to determine the ceiling and floor effects of scores.

We hypothesized the following:

(1) The test–retest reliability of SAQv1.1 scores would meet the COnsensus-based Standards for the selection of health status Measurement Instruments (COSMIN) [12] for measuring groups in research (ICC = 0.70) and for individual patient clinical use (ICC = 0.90), and would outperform the SAQ [13].
(2) The SAQv1.1 score (low is good) would show a negative correlation (≤−0.35) with the SRS-22r (high is good) [14]. The SAQv1.1 would show a stronger correlation with the SRS-22r Self-Image and would be equally or more strongly correlate with radiographic measurements.
(3) The SAQv1.1 scores would be free from ceiling and floor effects or have fewer such effects than the SAQ (<15%) [13].

These hypotheses were formulated because larger values for radiographic measurements have previously been shown to relate to worse HRQOL and perceived self-image [15,16].

## 2. Materials and Methods

This study includes a cross-sectional design to assess validity and ceiling and floor effects, as well as a repeated-measures design to evaluate reliability.

### 2.1. Participants

One hundred participants were recruited consecutively during clinic visits to a tertiary specialized pediatric scoliosis clinic. Participants (or their guardians) signed informed consent forms. Participants then completed the questionnaires before meeting their specialist. This study was approved by the Health Research Ethics Board at the University of Alberta. The inclusion criteria were as follows: females with a diagnosis of idiopathic scoliosis, aged 10 to 18 years, with a curve severity >10°, and fluent in English. While we did not set an upper limit of curve severity, the largest curve enrolled was 51°. All participants had been referred to the specialist for management of their scoliosis, which could include observation, exercises, bracing, or prescribing surgery. Exclusion criteria included having had a spine surgery, other conditions that impact HRQOL or torso appearance, or trauma involving the torso or lower extremities.

### 2.2. Data Collection

Participants were invited, using the Research Electronic Data Capture (REDCap) survey tool (version 7.6.9 [17]), to complete the questionnaires online during their clinic visit, and then again by automated invitations at one and two weeks post-visit. Study data were collected and managed using REDCap electronic data capture tools hosted by the Women & Children’s Health Research Institute at the University of Alberta. Up to three reminders, sent two days apart, were provided for each timepoint. Participants were reminded to enter any missing responses before submission. All questionnaires were completed in English.

Participants underwent a posterior–anterior frontal spine radiograph while standing, using the synchronous slot-scanning stereo-radiography system. Cobb angle, Axial Vertebral Rotation (AVR), and Coronal Balance measurements were performed by a single evaluator using MIAS (Medical Image Analysis Software, version 9.6.7.0, University of Alberta, Edmonton, AB, Canada). The evaluator, a physiotherapist, attended a demonstration, discussed ten practice cases, and measured practice cases twice (ICC_3,1_ = 0.99) to obtain Cobb angles. The evaluator remained blinded to the participants’ identities and questionnaire scores.

#### 2.2.1. Spinal Appearance Questionnaire (Original Version)

The SAQ evaluates patients’ perception of their appearance in relation to a spinal condition [7]. The SAQ includes 20 items, including eight pictograms illustrating spine-related postural issues [7]. Each pictogram has five drawings as response options, ranging from mild to severe (scores 1 to 5) [7]. The remaining 12 items ask about wanting to improve different posture elements, and they are answered by (1) ‘Not true’, (2) ‘A little true’, (3) ‘Somewhat true’, (4) ‘Fairly true’, or (5) ‘Very true’ [7]. Questions are summed to obtain nine domain scores (General (items #9, 10, and 19); Curve (#1); Prominence (#2 and 3); Trunk Shift (#4 and 5); Waist (#11, 12, and 13); Shoulders (#6 and 16); Kyphosis (#7); Chest (#14 and 15); and Surgical Scar (#17)) [7]. This SAQ scoring strategy does not include a total score. Sanders et al. demonstrated that the SAQ items have excellent test–retest reliability (Spearman’s rho = 0.57 to 0.99), and high internal consistency (Cronbach’s alpha > 0.70) [7].

#### 2.2.2. Spinal Appearance Questionnaire Version 1.1

Due to limitations with the original SAQ, Carreon et al. developed a modified version, the SAQv1.1, consisting of 14 items. The first ten items are presented as pictograms and pertain to the Appearance domain. The remaining four items are presented as text and contribute to the Expectations domain [11]. The Appearance items range from mild (score of 1) to severe (5). The items include the following: body curve; rib prominence; flank prominence; head–chest–hips alignment; position of head over hips; shoulder level; shoulder blade rotation; shoulder angle; head position; and spine prominence [11]. All Appearance items, except for three new items, appeared in the original version. Head position and shoulder rotation and angle were not in the original SAQ. These 10 items are summed up to provide the Appearance score, ranging from 10 to 50.

Four items form the Expectations domain: ‘want to be more even’; ‘to have more even shoulders’; ‘to have more even hips’; and ‘to have a more even waist’ [11]. These items’ text responses range from ‘not true’ (score of 1) to ‘very true’ (5). They are summed up to produce the Expectations score, ranging from 4 to 20 [11]. All four Expectations domain items were present in the original SAQ. The SAQv1.1 Total score is calculated as the sum of both domain scores and ranges from 14 (better perceived appearance) to 70 [11].

Carreon et al., in 1802 adolescents with mostly moderate to severe AIS, found good reliability (Cronbach’s α ≥ 0.88; test–retest correlation ≥ 0.81) and convergent validity with the major curve angle (r = 0.32; 0.36) for the SAQv1.1 [11].

#### 2.2.3. Scoliosis Research Society-22 Refined

The SRS-22r is the most widely used scoliosis-specific questionnaire assessing HRQOL [18]. It includes 22 questions spanning five domains: Pain, Self-Image, Function, Mental Health (each with five questions), and Satisfaction with Management (two questions). Each question is scored from 1 to 5 (best). Items are averaged to generate the domain and the Total score [18]. Higher SRS-22r scores reflect better HRQOL.

In surgically treated AIS, the SRS-22 previously demonstrated acceptable reliability (ICC = 0.85 to 0.96) and concurrent validity with the Short Form-36 (r > 0.70) [18]. However, 56.9% of respondents showed ceiling effects [18]. To enhance internal consistency in the Function domain, the SRS-22 was refined to produce the SRS-22r, resulting in an increase in the Cronbach α from 0.67 to 0.78 [19]. Despite these improvements, ceiling effects as high as 47% in the Function domain were still observed in Asher et al.’s refinement study [19].

#### 2.2.4. Cobb Angle

The Cobb angle is the frontal angle between the most tilted vertebra above and below the apex of each curve [20]. It is the most accurate and reliable method for quantifying scoliosis severity [21,22]. Cobb angle measurements were obtained using custom Medical Image Analysis Software (MIAS) version 9.6.7.0.

#### 2.2.5. Axial Vertebral Rotation

AVR is the rotation of a vertebra in the transverse plane [23]. Stokes’ method measures Vertebral Axial Rotation in degrees by calculating the distances between the centers of both pedicles and the vertebral center, while considering vertebral width and level [24]. Using MIAS, the evaluator first placed and adjusted the dimensions of pedicle landmarking ovals, then marked the lateral width limits of the vertebra at the narrowest point, and finally specified the vertebral level. MIAS then generated the rotation measurement [25]. Morrison et al. demonstrated excellent intra-rater reliability (ICC_2,1_ 0.83 to 0.99) and good inter-rater reliability (ICC_2,1_ > 0.83) for AVR measurements in AIS [26].

#### 2.2.6. Coronal Balance

Coronal Balance is the horizontal distance between vertical lines at the center of C7 and at the center of S1 [27]. Kuklo et al. demonstrated excellent intra- (ICC_2,1_ ≥ 0.98) and inter-evaluator reliability (ICC_2,1_ = 0.99) for Coronal Balance on radiographs in AIS [28].

### 2.3. Statistical Analysis

A descriptive analysis was conducted using the Statistical Package for the Social Sciences (SPSS) version 24.0 (SPSS Inc., Chicago, IL, USA). We also reviewed the pairwise scatter plots for the convergent validity and reliability analyses.

The Intraclass correlation coefficient (ICC_3,1_) was used for test–retest reliability using data collected at one and two weeks after the clinic visit. Week one and week two after the specialist consultation were deemed more likely to include an interval with clinical stability. This is because new treatments are rarely implemented so soon after the consultation. Further, this interval, even if a treatment had been implemented, would be unlikely to lead to observable changes. In contrast, since our baseline questionnaires were collected before the specialist consultation, it was possible that between baseline and the follow-up timepoints, discussions with the specialist may have influenced the questionnaire responses regarding perceived appearance. The Standard Error of Measurement (SEM) and the Minimum Detectable Change (MDC95 = SEM × 1.96 × √ 2) were also calculated [29]. Based on COSMIN recommendations, test–retest ICCs > 0.70 were deemed adequate for group analyses in research, and ICCs > 0.90 were deemed adequate for individual measurements clinically [13].

Pearson coefficients were used to quantify the correlation between questionnaire scores and radiographic measurements. A one-tailed significance level was used, as larger radiographic measurements often correlate with worse HRQOL and perceived self-image scores [15,16]. The threshold for the clinical importance of correlations assessing convergent validity was set at 0.35 or higher in the present study.

Ceiling and floor effects were calculated as the percentage of participants obtaining the best and worst possible scores, respectively [13]. The significance level was set at 0.05.

For test–retest reliability, having 42 participants, each with two observations, achieves > 80% power to detect an ICC of 0.85 as different from a reference ICC of 0.70 (or 0.90 > 0.795) with a significance level of 0.05. For validity, a sample of 100 participants achieves 80% power to detect a difference between a null hypothesis correlation of 0.35 and an alternative hypothesis of 0.57, also at a significance level of 0.05 (PASS 13. NCSS LLC., Kaysville, UT, USA).

## 3. Results

### 3.1. Participants

Participants included 100 females with a mean age of 13.9 ± 1.8 years and a Cobb angle of 28.8° ± 13.9°. In total, 38 participants were treated with bracing, 25 with exercise, and 37 were under observation. The 42 participants who completed the reliability study had Cobb angles of 25° ± 9° (Table 1).

### 3.2. Test–Retest Reliability (One to Two Weeks Post-Consultation)

The reliability sample had an SRS-22r Total score of 4.2 ± 0.5 and a SAQv1.1 Total score of 25.4 ± 8.4 (Table 2). All SAQ scores had test–retest reliability (ICC_3,1_ = 0.72 to 0.94) adequate for research (ICC_3,1_ > 0.70) (Table 3). However, only three of the eight tested SAQ scores had test–retest reliability adequate for individual use (ICC_3,1_ > 0.90). The SAQv1.1 Total and Appearance scores (ICC_3,1_ = 0.92 and 0.94, respectively) showed test–retest reliability adequate for research and individual use, while Expectations (ICC_3,1_ = 0.86) was adequate for research. SEMs were lower for SAQv1.1 Total and Appearance scores than for all SAQ domains (Table 3). The Total SRS-22r score demonstrated test–retest reliability adequate for both research and individual use (ICC_3,1_ = 0.94). SRS-22r domains had test–retest reliability ICC_3,1_ between 0.84 and 0.96 (Table 3).

### 3.3. Convergent Validity with the SRS-22r

The SAQ domains showed significant correlations with the SRS-22r Total: General (r = −0.59), Curve (−0.32), Prominence (−0.36), Trunk Shift (−0.40), Waist (−0.40), Shoulders (−0.35), Kyphosis (−0.35), and Chest (−0.52) (Table 4). All except Curve met the convergence threshold. Additionally, these SAQ domains correlated significantly with the SRS-22r Pain (r = −0.24 to −0.39), Function (−0.28; −0.44), Self-Image (−0.21; −0.70), and Satisfaction domains (−0.21; −0.33). However, five of the eight did not meet the convergence threshold with Pain, and four each did not meet the threshold with Function and Self-Image. No SAQ domains met the threshold with Satisfaction. Five SAQ domains also correlated with Mental Health (−0.19; −0.43), but only one met the convergence threshold.

The SAQv1.1 Total and domain scores also demonstrated significant correlations, with all meeting the convergence threshold with the SRS-22r Total (r = −0.44 to −0.50). The correlation between SAQv1.1 Appearance and SRS-22r Self-Image was also significant and met the threshold (r = −0.37) but was lower than that observed for three of the eight SAQ domains (General −0.70, Waist −0.43, and Chest −0.51). The correlations were significant between the SAQv1.1 scores and the SRS-22r Function, Pain, Mental Health, and Satisfaction domains. They ranged from r = −0.23 to −0.42. (Table 4). All three SAQv1.1 scores did not meet the convergence threshold with Mental Health, and the Expectations score also did not meet the threshold with Pain and Satisfaction.

### 3.4. Convergent Validity with Radiograph Measurements

For the SAQ domains, correlations ranged from r = 0.32 to 0.59 with the Cobb angle, 0.26 to 0.43 with Thoracic Rotation, and from not significant to 0.35 with Coronal Balance (Table 5). All but Chest met the convergence threshold with the Cobb angle. Only Trunk Shift and Chest met this threshold with Thoracic Rotation, and only Trunk Shift met it with Coronal Balance.

All correlations between the SAQv1.1 scores and the Cobb angle were significant and met the threshold, ranging from r = 0.38 for Expectations to r = 0.63 for Appearance. All correlations between SAQv1.1 scores and Thoracic Rotation were significant (r = 0.25 to 0.46), but Expectations did not meet the threshold. The correlations of SAQv1.1 scores with Coronal Balance ranged from not significant with Expectations to 0.35 with Appearance (only Expectations met the threshold). Low and non-significant correlations were found between all domains of both SAQ versions and Lumbar Rotation, except for the SAQ Curve domain, which reached significance and met the convergence threshold (r = 0.37) (Table 5).

### 3.5. Ceiling and Floor Effects

Ceiling effects were found in all domains of the SAQ and ranged from 11% for Curve to a high of 68% for Kyphosis. Floor effects were low and only observed for Waist (4%) and Chest (8%).

For the SAQv1.1, some low ceiling effects were observed in the Total (3%), Appearance (5%), and Expectations (14%) scores (Table 6). With SAQv1.1, only Expectations demonstrated floor effects, at 10%.

## 4. Discussion

While all original SAQ domains met the reliability threshold to be recommended for research (ICC > 0.70), only the General, Prominence, and Chest domains demonstrated sufficient test–retest reliability to meet the recommended standards for both research and individual use (ICC > 0.90). Sanders et al. previously reported similar test–retest reliability for individual SAQ items (Spearman’s rho = 0.57 to 0.99) [7]. As anticipated, in the present study, the SAQv1.1 Total and Appearance achieved adequate test–retest reliability for both research and individual use [13]. However, the Expectations domain only satisfied the threshold for research use (ICC_3,1_ = 0.86), potentially due to its lower number of items compared to the Appearance domain. The error estimates were also generally smaller for the SAQv1.1. These results differ from prior findings by Carreon et al. that the SAQv1.1 Total and Appearance scores did not satisfy the reliability threshold for individual use, while the Expectations domain did [11].

In the present study, both SAQ versions showed adequate convergent validity. However, not all SAQ domains reached the minimum convergence threshold (r ≥ 0.35) with the SRS-22r Total and Self-Image scores. In contrast, all SAQv1.1 scores met the hypothesized convergence validity threshold. This is possibly because the distribution of original SAQ scores relies on fewer items compared to the SAQv1.1. While COSMIN suggests aiming for correlations exceeding 0.70 when comparing to a gold standard criterion, they recommend adopting a lower threshold considering the conceptual differences between the constructs correlated to demonstrate convergent validity [12]. Because the SRS-22r domains and the radiographic changes used to test convergence do not reflect exactly the same construct as perceived appearance, as recommended by COSMIN, a lower convergence threshold (r ≥ 0.35) was adopted by team consensus specifically for this study [12]. Carreon et al. had found that only the SAQv1.1 Appearance domain met this convergence criterion with SRS-22 Self-Image (r = −0.39) [11]. The larger severity of Carreon et al.’s sample and their allowing parents’ input may explain the different results [11].

As hypothesized, all SAQv1.1 scores correlated more strongly with the SRS-22r Self-Image score than most (5/8) of the original SAQ scores. However, contrary to our hypotheses, three of the eight SAQ domains (General −0.70, Waist −0.43, and Chest −0.51) showed correlation estimates with the SRS-22r Self-Image score that were larger than with SAQv1.1 Appearance (−0.37), and two of these exceeded the corresponding correlations with SAQv1.1 Expectations and Total scores (both r = −0.46). Interestingly, these three scores are the only domains from the original SAQ that do not use pictograms in their scoring; the SAQv1.1 Appearance does. It is possible that the SRS-22r Self-Image construct is more closely related to the expectations or impact of self-image-related concerns than simply perceived appearance. The wording of two SAQ General items related to appearance in clothes and specifically self-image corresponds closely to items of the SRS-22r Self-Image, possibly explaining this higher correlation. Nevertheless, our second hypothesis of stronger correlations between SAQ v1.1 and SRS-22r Self-Image was partially met.

Our hypothesized convergence with the Cobb angle, an indicator of scoliosis severity, was not observed across all SAQ domains, while all SAQv1.1 scores demonstrated the expected convergence. The Cobb angle correlated most strongly with SAQv1.1 Appearance (r = 0.63). Thoracic Vertebral Rotation exhibited hypothesized levels of convergence with SAQv1.1 Total and Appearance scores, but most original SAQ domains did not. Coronal Balance only demonstrated convergence with SAQv1.1 Appearance. Once again, the fewer items contributing to the original SAQ scores and the milder severity of the sample in the present study may explain these results.

Lumbar rotation did not correlate with SAQ domains, except for Curve. This could be due to the low number of lumbar curves in our sample or, more likely, to the presence of soft tissue surrounding the lumbar spine, which can mask the effects of scoliosis. Indeed, Asher et al. observed similar findings showing stronger correlations between single thoracic curve magnitudes with SRS-22 function (r = −0.53) and Self-Image (r = −0.46), as well as between the surface topography Hump Index measurement and function (r = −0.60) [30]. In Asher et al.’s study, radiographic and surface topography measurements in double curves and thoracolumbar curves did not correlate significantly with SRS-22 responses [30]. Interestingly, in the present study, the SAQv1.1 had a stronger correlation with the Cobb angle than with the SRS-22r Self-Image and Total scores (other self-reported subjective measurements). This is consistent with our hypothesis, given that the SAQ focuses on perception of asymmetries while the SRS-22r addresses the related quality of life. Our results align with those of Carreon et al., who also observed a stronger correlation of the SAQv1.1 Total with curve magnitude (r = 0.32) than with the SRS-22 Self-Image and Total scores (r = −0.20 and −0.11) [11]. In summary, the convergent validity analyses performed between the SAQv1.1 and SRS-22 scores and the Cobb angle could be considered confirmatory, as we could compare them with the results from Carreon et al. [11]. However, convergent validity analyses of the SAQv1.1 compared to the other radiographic severity parameters are novel and represent exploratory analyses that should be replicated.

Consistent with Carreon et al. [11], the present study extends support for the SAQv1.1 for both clinical and research use in AIS in a sample consisting mostly of mild–moderate curves treated non-operatively. The SAQv1.1 may better detect changes compared to the original SAQ, as it exhibits fewer ceiling and floor effects. The smaller MDCs for the SAQv1.1 also suggest an improved capacity to detect small changes with confidence that they exceed measurement error. The presence of a ceiling effect can theoretically limit the ability to detect improvement because patients beginning a study with the best possible score cannot register an improvement on this questionnaire, even if the construct being measured had been perceived as improved by the participant. This could occur when questionnaires do not have items allowing for the detection of small changes in the better portion of the score and when scores are computed from too few items. Similarly, a floor effect could limit the ability to detect improvement for a patient whose status is worse than the worst score that can be registered by the tool. Such a patient could improve from that initial state to the state registered as the worst score on the tool and still show no change in score. Finally, a tool with a smaller detectable change due to smaller measurement error should allow more confidence in detecting smaller changes with confidence that they exceed measurement error. Although the SAQv1.1 Expectations domain showed acceptable ceiling effects, it did present the most ceiling effects among the SAQv1.1 scores. This could be due to its lower number of items. Alternatively, it is possible that the milder cases in our samples did not want to change their appearance.

Longitudinal responsiveness studies are needed to directly document the ability to detect change before recommending broader implementation. Only Sanders et al. have reported on responsiveness for the original SAQ in adolescents tested before and one year after surgery [7]. Notably, the SAQ had six domains showing a standardized response mean (SRM ≥ 1.0), while only the SRS-30 Appearance score reached this threshold [7]. To our knowledge, the responsiveness of the SAQv1.1. has not been studied yet.

The present study utilized Classical Test Theory to assess measurement properties. Item response theory could be used in the future to investigate scaling issues [13]. Additionally, other language versions could be assessed. The SAQ is available in English, Chinese, German, Spanish, Polish, Turkish, French, Swedish, Korean, and Danish [5]. Research in younger and older age groups, or other spinal conditions, or from pre- to post-operation is also needed.

The generalizability of the present study findings might be limited due to recruitment from a single specialized clinic and the exclusion of patients who had undergone surgery. However, we focused on patients treated conservatively as an understudied population where existing tools have previously been criticized for not performing adequately [4,10]. Additionally, since the present study was part of an analysis of multiple other questionnaires, many participants (58%) did not complete the retests, likely due to fatigue from repeated administration of five questionnaires within one week. If reliability is related to interest in repeated administration, our results may not be generalizable. Nonetheless, our test–retest comparisons were based on the same sample, allowing for the interpretation of relative performance, despite our reliability sample having slightly smaller curve angles than our larger validity sample.

## 5. Conclusions

The good measurement property results that we documented in females with mild–moderate AIS treated conservatively add to the promising results of Carreon et al. in a more severely affected sample [11]. Therefore, we support the use of the SAQv1.1 over the original SAQ in both research and clinical practice, as the SAQv1.1 demonstrates better measurement properties for a wide range of AIS severities. However, further evidence on the responsiveness of SAQv1.1 is needed before wide adoption.

## Figures and Tables

**Table 1:** Description of the participants.

	Validity Sample			Reliability Sample		
	n	Range	Mean (SD)	n	Range	Mean (SD)
Age (years)	100	10–18	13.9 (1.8)	42	10–17	13.5 (1.8)
Maximum Cobb angle (°)	99	10–74	29 (14)	41	10–51	25 (9)
Maximum Thoracic Rotation (°)	95	0.7–23.3	6.9 (4.3)	39	0.7–13.1	5.8 (3.4)
Maximum Lumbar Rotation (°)	82	1.0–20.0	7.6 (4.0)	35	1.3–16.2	7.1 (3.2)
Coronal Balance (mm)	99	0–22.1	7.2 (5.4)	41	0.2–22.0	7.2 (5.5)

n: Number of participants; SD: Standard deviation; °: Degree.

**Table 2:** Means and standard deviation for all questionnaires at all time points.

Questionnaire	Good HRQOL	Domains		Mean (SD)		
				Baseline (n = 100)	One Week (n = 42)	Two Weeks (n = 42)
SAQ	Low	General	/15	7.4 (3.2)	6.6 (3.2)	6.3 (3.2)
		Curve	/5	2.1 (0.7)	1.9 (0.5)	1.8 (0.4)
		Prominence	/10	3.3 (1.2)	3.2 (1.3)	3.1 (1.2)
		Trunk Shift	/10	3.4 (1.2)	3.3 (1.2)	3.1 (1.0)
		Waist	/15	6.6 (3.7)	5.8 (3.2)	5.6 (3.3)
		Shoulders	/10	4.5 (2.1)	4.3 (2.0)	4.3 (2.1)
		Kyphosis	/5	1.4 (0.7)	1.5 (0.7)	1.4 (0.6)
		Chest	/10	3.7 (2.5)	3.6 (2.3)	3.6 (2.5)
SAQv1.1	Low	Appearance	/50	17.6 (5.3)	17.2 (5.1)	16.2 (4.3)
		Expectations	/20	10.7 (5.4)	9.4 (4.8)	9.3 (5.3)
		Total	/70	28.2 (9.2)	25.4 (8.4)	25.5 (8.6)
SRS-22r	High	Function	/5	4.5 (0.5)	4.6 (0.4)	4.6 (0.4)
		Pain	/5	4.2 (0.8)	4.3 (0.7)	4.3 (0.7)
		Self-Image	/5	4.1 (0.7)	4.1 (0.7)	4.1 (0.8)
		Mental Health	/5	3.9 (0.7)	4.0 (0.7)	4.1 (0.6)
		Satisfaction	/5	3.9 (1.0)	4.2 (1.0)	4.2 (0.9)
		Total	/5	4.1 (0.5)	4.2 (0.5)	4.3 (0.5)

n: Number of participants, SRS-22r: Scoliosis Research Society-22 (refined), SAQ: Spinal Appearance Questionnaire, SD: Standard deviation, HRQOL: Health-related quality of life.

**Table 3:** Test–retest reliability of questionnaire scores.

Questionnaire	Good HRQOL	Domains		Test–Retest Reliability One vs. Two Weeks			
				n	ICC_3_,_1_ (95% CI)	SEM (% of Score)	MDC95 (% of Score)
SAQ	Low	General	/15	42	0.91 (0.84; 0.95)	1.0 (19.2)	2.7 (53.2)
		Curve	/5	42	0.79 (0.64; 0.88)	0.2 (4.4)	0.6 (12.2)
		Prominence	/10	42	0.90 (0.83; 0.95	0.4 (7.0)	1.0 (19.2)
		Trunk Shift	/10	42	0.89 (0.80; 0.94)	0.3 (6.6)	0.9 (18.4)
		Waist	/15	42	0.72 (0.53; 0.84)	1.7 (34.6)	4.8 (95.8)
		Shoulders	/10	42	0.85 (0.73; 0.91)	0.8 (16.6)	2.3 (45.8)
		Kyphosis	/5	42	0.82 (0.69; 0.90)	0.3 (5.6)	0.8 (15.4)
		Chest	/10	41	0.94 (0.89; 0.97)	0.6 (11.6)	1.6 (32.2)
SAQv1.1	Low	Appearance	/50	42	0.94 (0.89; 0.97)	1.1 (2.1)	3.0 (5.9)
		Expectations	/20	42	0.86 (0.75; 0.92)	1.9 (9.5)	5.3 (26.4)
		Total	/70	42	0.92 (0.87; 0.96)	2.3 (3.3)	6.3 (9.0)
SRS-22r	High	Function	/5	42	0.84 (0.73; 0.91)	0.2 (3.2)	0.5 (9.0)
		Pain	/5	42	0.96 (0.92; 0.98)	0.2 (3.0)	0.4 (8.6)
		Self-Image	/5	42	0.91 (0.83; 0.95)	0.2 (4.6)	0.6 (12.6)
		Mental Health	/5	42	0.91 (0.84; 0.95)	0.2 (3.8)	0.5 (10.6)
		Satisfaction	/5	42	0.88 (0.79; 0.93)	0.3 (3.6)	0.9 (18.2)
		Total	/5	42	0.94 (0.90; 0.97)	0.1 (2.4)	0.3 (6.6)

n: Number of participants, SRS-22r: Scoliosis Research Society-22 (refined), SAQ: Spinal Appearance Questionnaire, ICC: Intraclass Correlation Coefficient, SEM: Standard Error of Measurement, MDC95: Minimal Detectable Change estimated using 95% Confidence Interval, CI: Confidence Interval, HRQOL: Health-related quality of life.

**Table 4:** Correlation estimates for convergent validity between SAQ versions and SRS-22r scores.

Questionnaire	Domain		SRS-22r					
			Function	Pain	Self-Image	Mental Health	Satisfaction	Total
SAQ	General	r	−0.42 **	−0.32 **	−0.70 **	−0.43 **	−0.33 **	−0.59 **
	Curve	r	−0.28 **	−0.24 *	−0.26 *	−0.19 *	−0.24 *	−0.32 **
	Prominence	r	−0.30 **	−0.39 **	−0.26 **	−0.13	−0.30 **	−0.36 **
	Trunk Shift	r	−0.33 **	−0.34 **	−0.34 **	−0.24 *	−0.28 **	−0.40 **
	Waist	r	−0.36 **	−0.39 **	−0.43 **	−0.22 *	−0.28 **	−0.40 **
	Shoulders	r	−0.31 **	−0.30 **	−0.35 **	−0.13	−0.21 *	−0.35 **
	Kyphosis	r	−0.37 **	−0.32 **	−0.21 **	−0.13	−0.29 **⊥	−0.35 **
	Chest	r	−0.44 **	−0.35 **	−0.51 **	−0.34 **	−0.30 **⊥	−0.52 **
SAQv1.1	Appearance	r	−0.42 **	−0.41 **	−0.37 **	−0.24 *	−0.37 **	−0.48 **
	Expectations	r	−0.34 **	−0.32 **	−0.46 **	−0.23 *	−0.31 **	−0.44 **
	Total	r	−0.42 **	−0.40 **	−0.46 **	−0.26 **	−0.38 **	−0.50 **

r: Pearson correlation coefficient; SRS-22r: Scoliosis Research Society-22 (refined); SAQ: Spinal Appearance Questionnaire; Number of participants, 100 unless specified with; ⊥: 98 participants; ** Correlation is significant at the 0.01 level (2-tailed); * Correlation is significant at the 0.05 level (2-tailed).

**Table 5:** Correlation between SAQ domain scores from each version and radiographic measurements.

Questionnaire	Domain		Maximum Cobb Angle	Maximum Thoracic Rotation	Maximum Lumbar Rotation	Coronal Balance
SAQ	General	r	0.44 **	0.33 **	0.16	0.20
		n	99	95	82	99
	Curve	r	0.56 **	0.30 **	0.37 **	0.29 **
		n	99	95	82	99
	Prominence	r	0.51 **	0.34 **	0.10	0.33 **
		n	100	100	100	100
	Trunk Shift	r	0.59 **	0.43 **	0.16	0.35 **
		n	99	95	82	99
	Waist	r	0.38 **	0.26 *	0.14	0.25 *
		n	99	95	82	99
	Shoulders	r	0.40 **	0.30 **	0.05	0.08
		n	99	95	82	99
	Kyphosis	r	0.47 **	0.31 **	−0.03	0.31 **
		n	98	94	81	98
	Chest	r	0.32 **	0.40 **	0.04	0.26 *
		n	98	94	81	98
SAQv1.1	Appearance	r	0.63 **	0.46 **	0.19	0.35 **
		n	100	100	100	100
	Expectations	r	0.38 **	0.25 *	0.12	0.17
		n	99	95	82	99
	Total	r	0.56 **	0.40 **	0.17	0.29 **
		n	99	95	82	99

n: Number of participants, r: Pearson correlation coefficient, SAQ: Spinal Appearance Questionnaire. * Correlation is significant at the 0.05 level (2-tailed). ** Correlation is significant at the 0.01 level (2-tailed).

**Table 6:** Ceiling and floor effects for each questionnaire score.

Questionnaires	Good HRQOL	Domains	Ceiling (%)	Floor (%)
SAQ	Low	General	13	0
		Curve	11	0
		Prominence	33	0
		Trunk Shift	29	0
		Waist	30	4
		Shoulders	27	0
		Kyphosis	68	0
		Chest	56	8
SAQv1.1	Low	Appearance	5	0
		Expectations	14	10
		Total	3	0

SAQ: Spinal Appearance Questionnaire, HRQOL: Health-related quality of life.

## Data Availability

The original contributions presented in this study are included in the Supplementary Material (Table S1: Study data). Further inquiries can be directed to the corresponding author.

## Supplementary Materials

The following supporting information can be downloaded at: https://www.mdpi.com/article/10.3390/children13010087/s1, Table S1: Study data.

## Abbreviations

The following abbreviations are used in this manuscript:

°	Degree
α	Alpha
AIS	Adolescent idiopathic scoliosis
AVR	Axial Vertebral Rotation
CI	Confidence interval
COSMIN	COnsensus-based Standards for the selection of health status Measurement Instruments
HRQOL	Health-related quality of life
ICC	Intraclass correlation coefficient
MIAS	Medical image analysis software
MDC95	Minimum detectable change (95% confidence)
n	Number of participants
r	Pearson correlation coefficient
REDCap	Research Electronic Data Capture
SRS-22r	Scoliosis Research Society-22 (revised)
SD	Standard deviation
SEM	Standard error of measurement
SAQ	Spinal appearance questionnaire
WRVAS	Walter Reed Visual Assessment Scale
